# Prolonged Neuromuscular Blockade Following Succinylcholine Administration to a Patient with a Reduced Butyrylcholinesterase Activity

**DOI:** 10.1155/2010/472389

**Published:** 2010-06-17

**Authors:** Ivo F. Panhuizen, Marc M. J. Snoeck, Soledad Levano, Thierry Girard

**Affiliations:** ^1^Department for Anaesthesiology, Canisius-Wilhelmina Hospital, C40-01 Postbus 9015, 6500 GS Nijmegen, The Netherlands; ^2^Department for Anaesthesiology, University Hospital Basel 4301 Basel, Switzerland

## Abstract

This case is about a 48-year-old woman known with a reduced butyrylcholinesterase activity, who developed prolonged neuromuscular blockade following the unintentional administration of succinylcholine. We took the opportunity to monitor the development of neuromuscular function during this period and blood samples were taken for molecular genetic analysis and for quantitative and qualitative analysis since not all causative mutations are functionally characterized. Reduced butyrylcholinesterase activity is discussed in many aspects. Clinical considerations are suggested concerning genetic counselling.

## 1. Introduction

Normally 90 percent of intravenously administered succinylcholine is hydrolysed in the plasma by butyrylcholinesterase (BCHe) before it can bind to the alpha subunits of the nicotinic cholinergic receptor at the neuromuscular junction. BCHe hydrolyses ester bonds in many agents; local anaesthetics-like cocaine, procaine, chloroprocaine, and tetracaine, and also heroine and mivacurium, are metabolised by BCHE. Decreased activity of BCHe becomes clinically relevant in patients carrying at least one causative mutation in the BCHE gene [1]. The BCHE gene has been identified on chromosome 3q26 [2]. The mechanism of decreased BCHe activity can be qualitative or quantitative, so different variants (phenotypes) of BCHe can be distinguished. Apart from the wild type (wt), many variants are known, for instance the atypical (A), silent (S), or fluoride-resistant (F) variant, as well as the quantitative J-, K- and H-variants [1]. 

Following administration of succinylcholine to a patient with a known abnormal variant of BCHe, the duration of relaxation of skeletal muscles is predictable. In this case history the genotype of the patient was not known yet. 

After informed consent obtained from the patient we describe this case history concerning prolonged neuromuscular blockade following succinylcholine administration to a patient with a reduced butyrylcholinesterase activity and the follow-up investigation.

## 2. Case Description

A 48-year-old woman (weight: 108 kg, height-170 cm) was scheduled for a laparoscopic cholecystectomy because of cholelithiasis. At the preoperative assessment she reported postoperative apnoea following a Caldwell-Luc procedure at the ENT-OR in 1981. A prolonged effect of succinylcholine had been confirmed by a dibucaine number of 10, written on a crumpled warning card. The patient herself was absolutely clear about the condition of her butyrylcholinesterase deficiency. Her medical history showed an appendectomy, hypertension, and a trigeminal neuralgia due to repetitive sinus infections. Current medication was amitriptyline, gabapentin, diclofenac, omeprazole, metoprolol, and acetaminophen combined with codeine. 

Induction of anaesthesia was carried out with fentanyl and propofol. To facilitate tracheal intubation 100 mg succinylcholine (0.9 mg/kg) had been administered unintentionally. Maintenance was performed with intermittent dosages of fentanyl, morphine and a continuous infusion of propofol. The surgical procedure lasted 90 minutes and was done uneventfully.

Monitoring of neuromuscular function was started using acceleromyography (TOF-Watch SX), by performing intermittently a “train-of-four”. Determination of the supramaximal stimulus could not be performed, as deep neuromuscular blockade was already induced. Anaesthesia, using propofol continuously in combination with intermittent doses of morphine, and neuromuscular monitoring were continued until recovery of the TOF-ratio above 90%; at this value it was assumed that the patient could breathe without help. Recovery of neuromuscular function took more than seven hours. The first twitch was seen 87 minutes after the administration of succinylcholine. The course was uneventful although the patient was emotional and anxious postoperatively we were able to regain her trust after several talks.

After informing this adverse event to the patient and her partner, she gave permission to take blood samples for quantitative and qualitative analysis and for molecular genetic investigation.

The plasma concentration of cholinesterase was 2520 U/l (ref: 4800–12000 U/l) and the dibucaine number was 8. These results are indicative for a homozygous atypical variant. Because of the very long clinical duration of action we did not limit molecular genetic investigations to known BCHE variants but decided to sequence both strands of the complete BCHE coding sequence. This identified a homozygous A- as well as a homozygous K-variant. The A-variant (209A > T, leading to Asp70Val) is most causative for the clinical feature and the K-variant (1615G > A, leading to Ala539Thr) contributed to the markedly prolonged muscle relaxation and the dibucaine number of 8. The clinical significance of the K-variant as a single mutation has been described by Gätke et al. [3].

## 3. Discussion

Immediately after induction, the attending anaesthesiologist realized that he had made a big mistake most probably caused by overconcentration. Although all preparations were done, in word and writing, this case shows us that anesthesia is done by humans. Unfortunately man makes mistakes. The best thing you can do afterwards is to be honest to the patient and that you will try to avoid it in the future.

Succinylcholine in a dosage of 1 mg/kg has a rapid onset (30–60 sec.) and a short duration of action (3–5 min.). It mimics the action of acetylcholine at the neuromuscular junction by depolarising the postjunctional membrane, provided that the depolarisation is sustained until succinylcholine is hydrolysed by BCHe. 

BCHe is synthesised by the liver, and among other plasma cholinesterases its physiological function is still not fully understood. In 1957, Werner Kalow identified plasma cholinesterase (pseudocholinesterase) as the cause of variable metabolism of drugs when he described persisting neuromuscular blockade (apnoea) after the administration of succinylcholine [4]. By investigating not only the plasma from susceptible patients but also from their parents, he discovered a genetic variant of BCHe. By this, he is one of the founder researchers of pharmacogenetics.

Genetic variants of butyrylcholinesterase result in differences in clinical response to succinylcholine. This often unexpected drug effect with potential morbidity as a consequence makes decreased BCHe activity a pharmacogenetic disorder [1]. The activity of the BCHe variants is expressed by a dibucaine number; the normal enzyme (usual variant: U) shows a dibucaine number of 70 (70% of its activity can be inhibited by dibucaine). The most often occurring abnormal enzyme is called the A-variant (atypical variant) which has a dibucaine number of 30 or less. Its prevalence is not rare since the allelic frequency of this A-variant is about 0.02, leading to an incidence of homozygotes (with a dibucaine number of 30 or less and a markedly clinical response) of 1 in 2500. Manifestation of the disorder presents during general anaesthesia where succinylcholine or mivacurium is used. The normal duration of neuromuscular blockade after administration of these agents is extended by hours. Cerf et al. described duration of paralysis ranging from 40 to 720 minutes [2]. Duration of neuromuscular blockade can be further increased by external (nongenetic) factors. These factors include, for instance, temperature, head- and neck-related malignancies, high oestrogen levels in parturients at term, and medication-like depakine or metoclopramide [2]. Patients with an abnormal BCHe activity who are given a normal dose of succinylcholine undergo a non-depolarizing-like block characterized by fade in the response to TOF which is also known as a phase II block.

In response to an adverse event in which an abnormal BCHe activity is suspected the first step is to perform biochemical investigations to confirm the phenotype [2]. To specify the decreased activity of BCHe molecular genetic diagnostics are available [5]. Only molecular genetic analyses are able to distinguish an acquired from an inherited BCHe deficiency [5]. Table 1 shows a summary of identified mutations in the BCHE gene, single nucleotide polymorphisms leading to phenotypical BCHe activity variants. DNA analysis in our patient led to the identification of 2 homozygous mutations. Linkage between the A- and K- variants has already been described by Bartels et al.; they found in 17 out of 19 genes bearing the A- variant also the K- variant [6].

The A- variant we detected (209A > T, leading to Asp70Val) is different from the most frequent occurring A variant (209A > T, leading to Asp70Gly). Diagnosing the mutations in the BCHE gene in our patient, we could offer family members genetic counselling to screen for this mutation [7]. To individuals carrying one or more of these causative mutations it might be advised to wear a medical alert saying that succinylcholine should not be administered. Unfortunately this case report shows that it cannot be guaranteed that succinylcholine will not actually be used. 

In conclusion, using neuromuscular blocking agents, it is recommended to monitor the recovery of neuromuscular function. Persisting neuromuscular blockade following the administration of succinylcholine should be investigated using conventional biochemical testing and molecular genetic testing to distinguish between an inherited or acquired cause for the decreased activity of BCHe. Applying this knowledge into clinical practice will improve the safety of our patients and their relatives. 

## Figures and Tables

**Table 1:** Genotype—Phenotype Relation of Butyrylcholinesterase (BCHE).

Nucleotide Exchange	Amino Acid Change	Phenotype Variants	Reference
98A > *G*	Tyr-33-Cys	Silent	[1]
109C > *T*	Pro-37-Ser	Silent	[1]
209A > *G*	Asp-70-Gly	Atypical	[2]
350T > *A* *G*	Gly-117-Gly+	Silent	[3]
355C > *T*	Gln-119-STOP	Silent	[4]
375A > *T*	Leu-125-Phe	Silent	[1]
383A > *G*	Tyr-128-Cys	Silent	[5]
424G > *A*	Val-142-Met	H	[6]
510T > *G*	Asp-170-Glu	Silent	[1]
551C > *T*	Ala-184-Val	Fluoride-Resistant	[7, 8]
592A > *G*	Ser-198-Gly	Silent	[1]
596C > *T*	Ala-199-Val	Silent	[9]
601G > *A*	Ala-201-Thr	Silent	[1]
728C > *T*	Thr-243-Met	Fluoride-Resistant	[10]
811G > *T*	Glu-271-STOP	Silent	[1]
1093G > *A*	Gly-365-Arg	Silent	[11]
1169G > *T*	Gly-390-Val	Fluoride-Resistant	[10]
1294G > *T*	Glu-432-STOP		[7]
1411T > *C*	Trp-471-Arg	Silent	[1]
1490A > *T*	Glu-497-Val	J	[12]
1500T > *A*	Tyr-500-STOP	Silent	[1]
1543C > *T*	Arg-515-Cys	Silent	[11]
1553A > *T*	Gln-518-Leu	Silent	[1]
1615G > *A*	Ala-539-Thr	K	[13]
